# Correction: Almutairi et al. The Expression Patterns of Human Cancer-Testis Genes Are Induced through Epigenetic Drugs in Colon Cancer Cells. *Pharmaceuticals* 2022, *15*, 1319

**DOI:** 10.3390/ph19010082

**Published:** 2025-12-31

**Authors:** Mikhlid H. Almutairi, Turki M. Alrubie, Bader O. Almutairi, Abdullah M. Alamri, Abdulwahed F. Alrefaei, Maha M. Arafah, Mohammad Alanazi, Abdelhabib Semlali

**Affiliations:** 1 Zoology Department, College of Science, King Saud University, Riyadh 11451, Saudi Arabia; 442106519@student.ksu.edu.sa (T.M.A.); bomotairi@ksu.edu.sa (B.O.A.); afrefaei@ksu.edu.sa (A.F.A.); 2 Genome Research Chair, Department of Biochemistry, College of Science, King Saud University, Riyadh 11451, Saudi Arabia; abdullah@ksu.edu.sa (A.M.A.); msanazi@ksu.edu.sa (M.A.); 3 Pathology Department, College of Medicine, King Saud University, Riyadh 11451, Saudi Arabia; marafah@ksu.edu.sa; 4 Groupe de Recherche en Écologie Buccale, Faculté de Médecine Dentaire, Université Laval, 2420 Rue de la Terrasse, Local 1758, Québec, QC G1V 0A6, Canada; abdelhabib.semlali@greb.ulaval.ca

## Error in Figure

In the original publication [1], there were mistakes in Figures 1 and 6. Specifically, the *ACTB* gene expression data for Caco-2 cells treated with 5-AZA for 48 h and HCT116 cells treated with 5-AZA for 72 h were incorrectly presented using the same gel image, despite differing experimental conditions. The error occurred in the gel image for *ACTB* in HCT116 cells treated with 5-AZA for 72 h. The corrected gel for *ACTB* in HCT116 cells treated with 5-AZA for 72 h has been updated in Figure 1 and Figure 6 and appears as shown below. The authors state that the scientific conclusions are unaffected. This correction was approved by the Academic Editor. The original publication has also been updated.

## Figures and Tables

**Figure 1:**
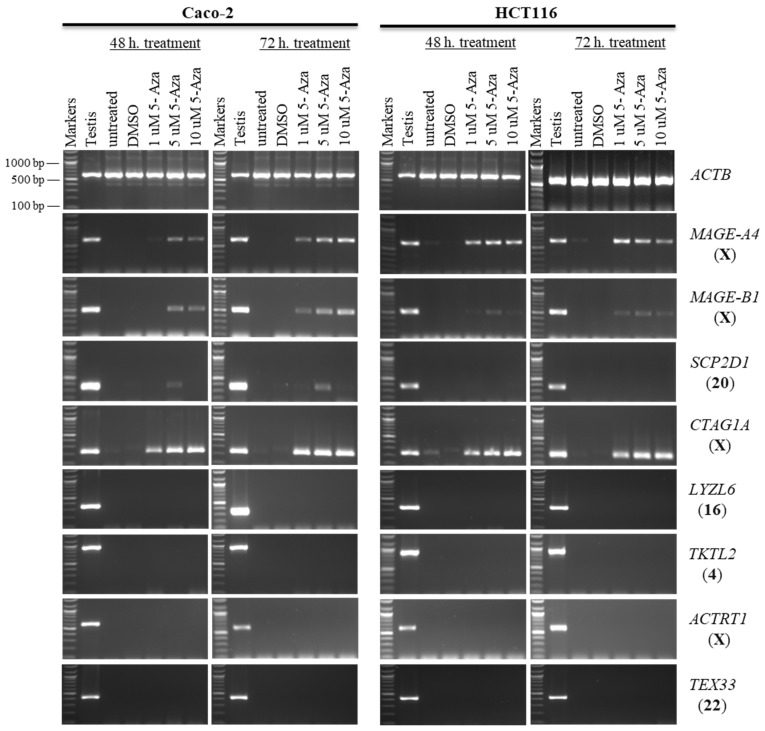
The effects of 5-aza-CdR treatments on CT gene expression profiles in the Caco-2 and HCT116 cancer cell lines. The expression of *SCP2D1*, *CTAG1A*, *LYZL6*, *TKTL2*, *ACTRT1*, and *TEX33* genes is shown on agarose gels following treatment with various doses of 5-aza-CdR for 48 h (left column of each cell) or 72 h (right column of each cell). Untreated Caco-2 and HCT116 cells were utilized as controls to compare the expression of CT genes with treated cells, and a testis sample served as a positive control for primer efficiency. The control Caco-2 and HCT116 cells were treated with DMSO, as DMSO was the solvent used in the 5-aza-CdR solution. The positive control for the cDNA samples is the expression of the *ACTB* gene. The official names of the genes are written to the right of the agarose gel images, and the location of each gene on the chromosomal is written in parentheses on the right. Above each lane, the particular concentration of 5-aza-CdR (1, 5, and 10 µM) is written.

**Figure 6:**
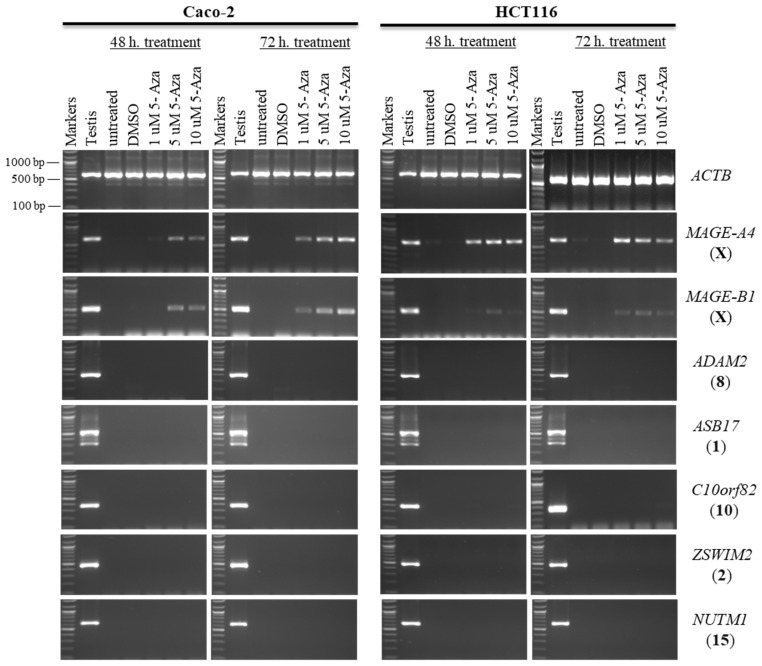
The effects of 5-aza-CdR treatments on testis-specific gene expression profiles in the Caco-2 and HCT116 cancer cell lines. The expression of *ADAM2*, *ASB17*, *C10orf82*, *ZSWIM2*, and *NUTM1* genes is shown on agarose gels following treatment with various doses of 5-aza-CdR for 48 h (left column of each cell) or 72 h (right column of each cell). Untreated Caco-2 and HCT116 cells were used as controls to compare the expression of CT genes with treated cells, and a testis sample served as a positive control for primer efficiency. The control Caco-2 and HCT116 cells were treated with DMSO, as DMSO was the solvent used in the 5-aza-CdR solution. The positive control for the cDNA samples is the expression of the *ACTB* gene. The official names of the genes are written to the right of the agarose gel images, and the location of each gene on the chromosomal is written in parentheses on the right. Above each lane, the particular concentration of 5-aza-CdR (1, 5, and 10 µM) is written.

## References

[1] Almutairi M.H., Alrubie T.M., Almutairi B.O., Alamri A.M., Alrefaei A.F., Arafah M.M., Alanazi M., Semlali A. (2022). The Expression Patterns of Human Cancer-Testis Genes Are Induced through Epigenetic Drugs in Colon Cancer Cells. Pharmaceuticals.

